# Skin cutaneous melanoma properties of immune-related lncRNAs identifying potential prognostic biomarkers

**DOI:** 10.18632/aging.203982

**Published:** 2022-03-31

**Authors:** Yutong Ma, Ning Wang, Shude Yang

**Affiliations:** 1 Department of Plastic Surgery, The First Hospital of China Medical University, Shenyang 110001, Liaoning Province, P.R. China; 2 Liaoning Provincial Key Laboratory of Oral Diseases, School of Stomatology, China Medical University, Shenyang 110001, Liaoning Province, P.R. China; 3 Department of Breast Surgery, The First Hospital of China Medical University, Shenyang 110001, Liaoning Province, P.R. China

**Keywords:** immune-related lncRNAs, prognostic risk, skin cutaneous melanoma, immune biomarkers, immune regulation

## Abstract

Skin cutaneous melanoma (SKCM) is one of the most aggressive and life-threatening cancers with high incidence rate, metastasis rate and mortality. Early detection and stratification of risk assessment are essential to treat SKCM and to improve survival rate. The aim of this study is to construct an immune-related lncRNAs (immlncRNAs) prognosis risk model to identify immune biomarkers for early diagnosis, prognosis assessment and target immunotherapy of SKCM. For this purpose, we identified 46 immlncRNAs significantly correlated with SKCM prognosis to construct the prognostic risk model and patients were stratified into the high- and low-risk subgroups according to the developed model. The predictive efficiency of this model has been proved by K-M survival analysis and receiver operating characteristic curve. Moreover, CIBERSORT algorithms confirmed that there were differences in immune cell infiltration between the high- and low-risk groups. Functional enrichment analysis further indicated that immlncRNAs were related to a variety of immune response process signaling pathways, suggesting that relevant immlncRNAs could play an important role in the immune regulation of SKCM. Finally, subgroup analysis and multiple Cox regression analysis further proved the stability of the model. In summary, we successfully constructed a 46 immlncRNA-related prognostic risk score model with excellent predictive efficacy and provided more possibilities to investigate the immune regulation mechanisms and to develop immunotherapy of SKCM.

## INTRODUCTION

Skin cutaneous melanoma (SKCM) is one of the most aggressive and life-threatening cancers [1]. The number of the new cases of cutaneous melanoma is increasing in recent years. Moreover, the prognosis is poor due to early metastasis which is the main cause of death of malignant skin tumors [2]. Therefore, early detection of SKCM and stratification of risk assessment are essential to treat SKCM and to improve survival rate.

Many studies reveal that misregulation of gene expression program is a key mediator in SKCM [3–5]. Long non-coding RNAs (lncRNAs) are defined as a type of RNA that is longer than 200 nucleotides and not translated into protein [6]. Considering that lncRNAs contain various transcripts, all of them can modulate gene expression in specific ways on a basis of cell type, developmental stage and function [7, 8]. lncRNAs play major roles in misregulation of gene expression [7, 8]. Despite a sea of previous works focusing on the function of lncRNAs and their mechanisms of action, the roles of lncRNAs in SKCM still remain elusive [9, 10]. Early studies have primarily analyzed that major possible influential factors of lncRNAs for SKCM development and progression often occur in the process of cell proliferation, apoptosis and differentiation, such as survival-associated mitochondrial melanoma–specific onco-genic non-coding RNA (SAMMSON) and SRA-like non-coding RNA (SLNCR1) [11–14].

Furthermore, as one of the most immunogenic tumors, the role of immune regulation and immunotherapy of SKCM are always the central issues [15]. More extended studies have identified tumor microenvironment as an important mediator associated with SKCM [16]. Evidences from Dummer’s and Bruno’s studies showed that dysregulation of the immune system could be a main cause of SKCM. And as a promising KCM treatment strategy, immunotherapy has triggered considerable recent research interest [17, 18]. Hence, precise regulation of immune genes expression leading to build a robust immune system does count. Whereas mechanisms of immune-related coding gene regulation have been extensively reported [19, 20], less is known about the regulation mechanisms of immune-related lncRNAs (immlncRNAs) in SKCM. Several known immlncRNAs have been proved to serve as a significant part in SKCM so far [21]. For example, the lncRNA THRIL regulates TNF-α release and global gene expression in human monocytic THP-1 cells [22]. The lncRNA SAMMSON knockdown drastically decreases the viability of melanoma cells irrespective of their transcriptional cell state and BRAF, NRAS or TP53 mutational status [11]. Therefore, further studies on lncRNAs and their roles in immune regulation are warranted to identify immunotherapy targets in SKCM.

In this study, we systematically identified immlncRNAs that were involved in SKCM, created and further validated prognostic risk model of SKCM. Overall, we propose that exploration of SKCM- related immlncRNAs reveals formation mechanism, immune regulation mechanism and immunotherapy of SKCM, as well as lays a solid foundation on evaluating the clinical prognosis of SKCM.

## RESULTS

### Preparation of SKCM data sets

First, a total of 472 SKCM samples including expression data of related 60498 genes were downloaded from TCGA database. At the same time, preliminary statistics on the phenotypic data of these samples was performed, turning out that the number of paracancer samples was far less than that of cancer samples (1:47.1). Therefore, expression data of normal samples were downloaded from GTEx database, and expression matrix of skin samples were extracted to successfully identify the differential expressed lncRNAs. Gene expression data of 471 cancer samples and 555 normal samples were finally obtained after integration. After that, a total of 14,081 expressed lncRNAs were obtained, by integrating gene expression data.

Next, the immlncRNA information was downloaded from the immLnc database, and a total of 12,499 immlncRNAs were obtained.

### Identification of differentially expressed lncRNAs (DE-lncRNAs) in SKCM

**Figure 1:**
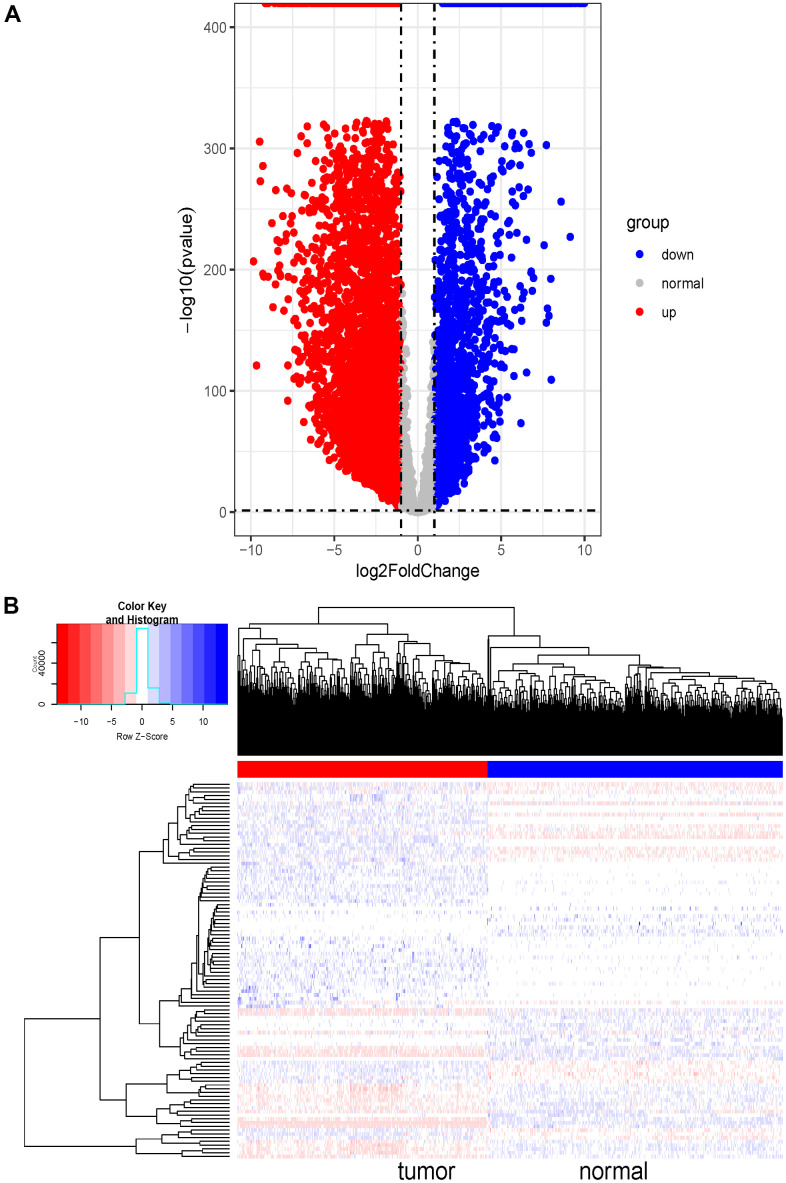
**Differential expression analysis and heatmap of DE-lncRNAs in SKCM.** (**A**) Volcano plot of 7836 DE-lncRNAs between SKCM and normal tissues. The Volcano plot was drawn with -log10 (p-value) as the vertical axis and log2 (Fold Change) as the horizontal axis. The dotted line was the threshold line. The horizontal dotted line represented p-value=0.05, and the vertical dotted line represented logFC<-1 and logFC>1. The red dots represented significantly upregulated DE-lncRNAs and the blue dots represented significantly downregulated DE-lncRNAs in SKCM tissues. The grey dots represented genes that were not differentially expressed. (**B**) Heat map of randomly selected 100 DE-lncRNA genes between SKCM and normal tissues. The red and blue bars represented the tumor samples and normal samples, respectively. And the evolution from red to blue represented the expression level of genes. The bluer zones indicated higher expression while the redder zones indicated lower expression.

The MDS (Multidimensional Scaling) diagram showed that the cluster of homogeneous samples was obvious and the difference of heterogeneous samples was significant. Based on the threshold abs(log_2_FC) > 1 and FDR < 0.05, 7836, DE-lncRNAs were screened out, among which 5637 were upregulated and 2199 were downregulated (Figure 1A). Moreover, 100 DE-lncRNAs were randomly selected to draw a heat map shown in Figure 1B (in the form of log_2_(x+1) for plotting data). The heat map clearly showed that the expressions of these DE-lncRNAs were indeed different between normal samples and tumor samples. The standardized data were extracted for subsequent weighted co-expression network analysis.

### The integration of DE-immlncRNAs

**Figure 2:**
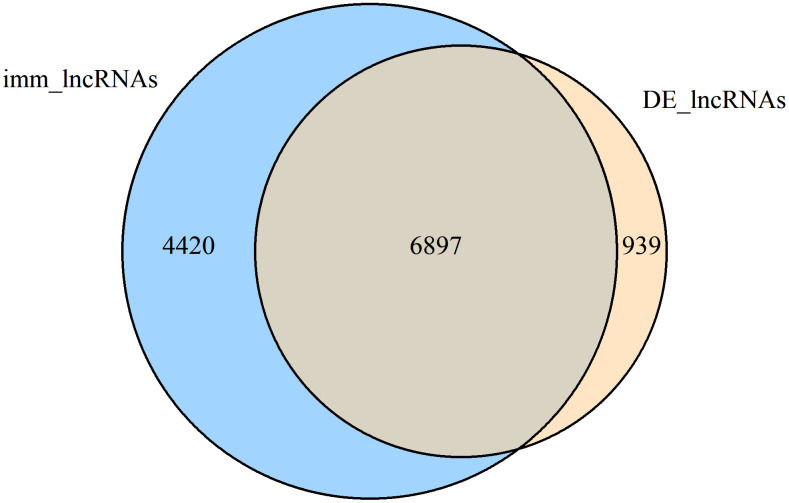
Venn diagram showing the number of common lncRNAs within DE-lncRNAs and immlncRNAs (DE-immlncRNAs).

We further integrated the shared DE-lncRNAs (7836) and immlncRNAs (12,499), and obtained the overlapping part shown in Figure 2 as DE-immlncRNAs (6,897) for subsequent WGCNA analysis.

### WGCNA analysis

**Figure 3:**
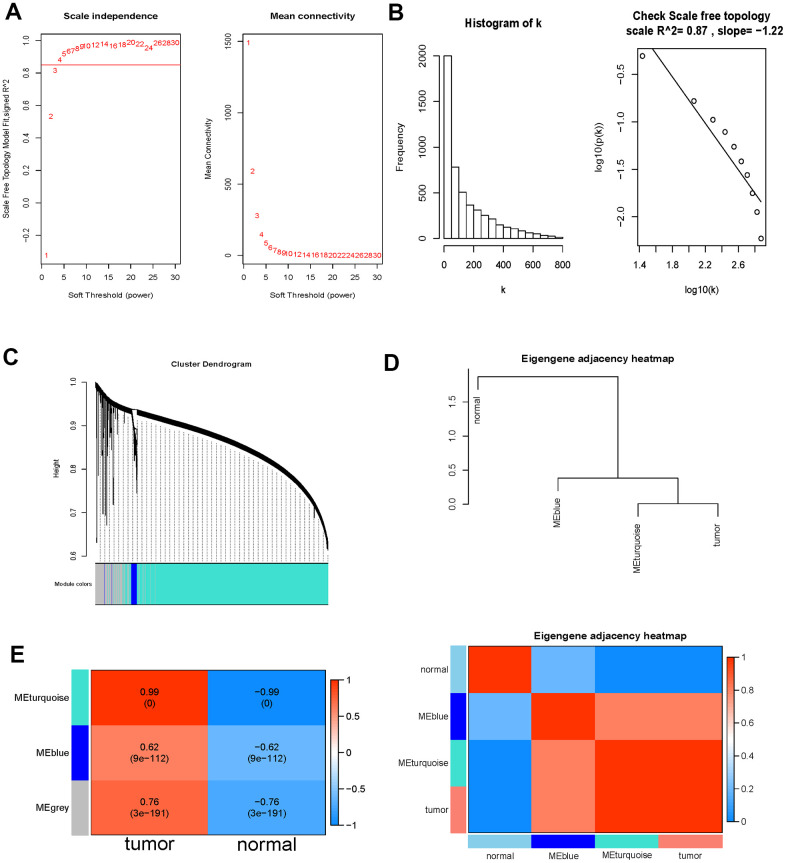
**WGCNA analysis outcome.** (**A**) Soft threshold filtering. The left diagram showed the relationship between the filtering threshold R^2 and the soft-thresholding power. The horizontal solid line indicated the screening threshold R2>0.85. And the right diagram showed the association between the mean connectivity and the soft-thresholding power. (**B**) Detection of the soft threshold of the network. The sloping solid line represented the fitted curve. There was a negative correlation between K and p(k) (correlation coefficient 0.87), indicating that the selected power value could establish a scale-free network. (**C**) Cluster dendrogram of the DE-immlncRNAs. Each branch on the upper side of the figure represented a lncRNA; On the bottom side of the figure, the attribution of lncRNA was marked corresponding to that on the top side, and each color represented a module. (**D**) Phenotypic dependent inter-module cluster diagram to visualize the relationships between different modules and phenotypes. (**E**) Heat map of correlation between the immlncRNA module and clinical phenotype. The value of the correlation coefficient decreased continuously from red to blue, and the redder zones indicated stronger positive correlation while the bluer zones indicated stronger negative correlation.

WGCNA analysis was carried out on 6897 DE-immlncRNAs. First, log_2_(x+1) transformation was performed on the analysis data. Then, 5079 lncRNAs with the first 75% median absolute deviation and MAD at least greater than 0.01 were screened for subsequent analysis. The soft-thresholding power was determined as 4 (power=4) based on the filtering threshold R^2 > 0.85 (Figure 3A). To ensure a scale-free network, the selected power value was tested. There was a negative correlation between K and p(k) (correlation coefficient 0.87), indicating that the selected power value could establish a scale-free network (Figure 3B). The network was constructed by one-step method with Power =4, and similar modules were merged with height < 0.25 as the threshold. In the end, only two modules were identified, turquoise=4248, Blue =138, unclassified (grey)=693 (Figure 3C). In order to further identify the key module with strongest correlation with SKCM, the correlation diagram between modules was drawn by combining phenotypic data. The results showed that turquoise module was clustered with tumor phenotype (Figure 3D). The correlation analysis of module and phenotype data further indicates that turquoise module has a high correlation with tumor with a correlation coefficient of 0.99 (P-value < 0.0001), which further indicates that lncRNAs in this module have a stronger correlation with SKCM, so lncRNAs in this module were selected for subsequent analysis (Figure 3E).

### Univariate Cox regression analysis of lncRNAs in key modules

**Figure 4:**
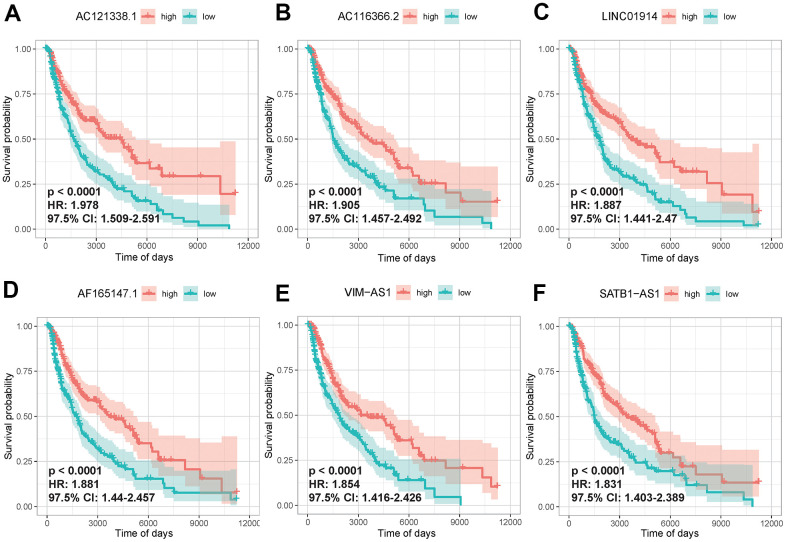
**Univariate Cox regression analysis of lncRNAs in key modules.** The K-M curves of the first 6 lncRNAs that were significantly correlated with disease prognosis, AC121338.1 (**A**), AC116366.2.C (**B**), LINC01914 (**C**), AF165147.1 (**D**), VIM-AS1 (**E**), SATB1-AS1 (**F**). High and low expression was shown in red and green, respectively.

To identify immlncRNAs associated with SKCM prognosis, 457 cancer samples which provided valid survival data were collated from the initial 471 cancer samples downloaded from TCGA for subsequent analysis. On this basis, batch univariate Cox regression analysis was carried out for 4248 lncRNAs in turquoise module. A set of 721 immlncRNAs in turquoise module were identified to have a significant association with prognosis of SKCM (P < 0.05). The lncRNAs in the first 6 positive phase relation values were further selected to draw the K-M curve to confirm the univariate Cox regression analysis results. The higher expression level of lncRNAs in the curve had a significant positive impact on survival (P-value < 0.0001), Hazard ratio was all greater than 1.8, and the interval of 97.5%Cl was greater than 1, indicating that univariate Cox regression analysis results were reliable (Figure 4).

### Establishment of immlncRNAs prognosis risk scoring model

**Figure 5:**
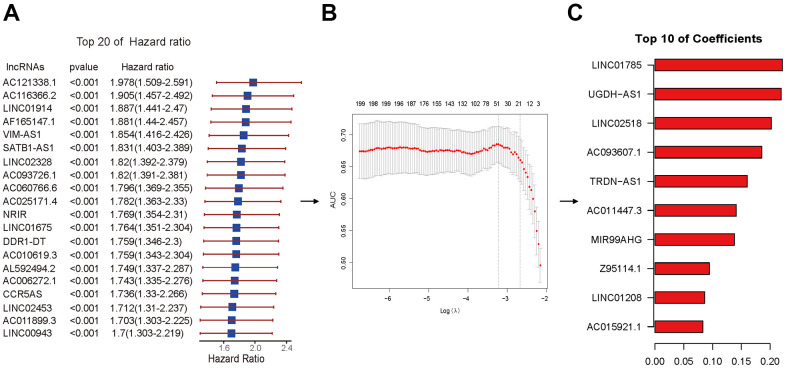
**Lasso regression performed by Cox regression results.** (**A**) The HR and p-value of selected lncRNAs with the top 20 of HR using the univariable Cox HR regression (p <0.001). (**B**) Cross-validation determined the optimal value of the penalty parameter (Lambda.min). Partial likelihood deviation curves were plotted against lambda. Dotted vertical lines were drawn at the optimal values by using the minimum criterion (left) and 1 standard error of the minimum criterion (1-SE criterion) (right). (**C**) The coefficients of selected lncRNAs with the top 10 of coefficients.

Lasso regression dimension reduction was ulteriorly performed on 721 lncRNAs with significant correlation with prognosis identified by univariate Cox regression analysis**.** The method of cross-validation is first used to identify the minimum lambda value namely Lambda.min with the purpose of building the best model. Optimum 46 ImmlncRNA with more significant prognostic value were filtered out under the Lambda.min to establish an immlncRNAs prognosis risk scoring model (Figure 5). To later verify the prediction effectiveness of the model more comprehensively, we cut data via R package caret and obtained 320 training sets and 137 testing sets considering there were lack of lncRNAs-related data sets and no suitable external data sets were found. Moreover, in the process of model construction, the code set.seed (1) was used to set the seed as 1 to realize the reproducibility of results. Then, the risk score (-0.2055-4.3401) of each sample in training sets was further calculated by using the formula in the method. The 320 samples were divided into high- and low-risk groups using the median (1.9550) as the cutoff point, and 160 of the high- and low-risk groups were obtained respectively for subsequent analysis.

### Prediction effectiveness analysis and verification of prognosis risk scoring model

**Figure 6:**
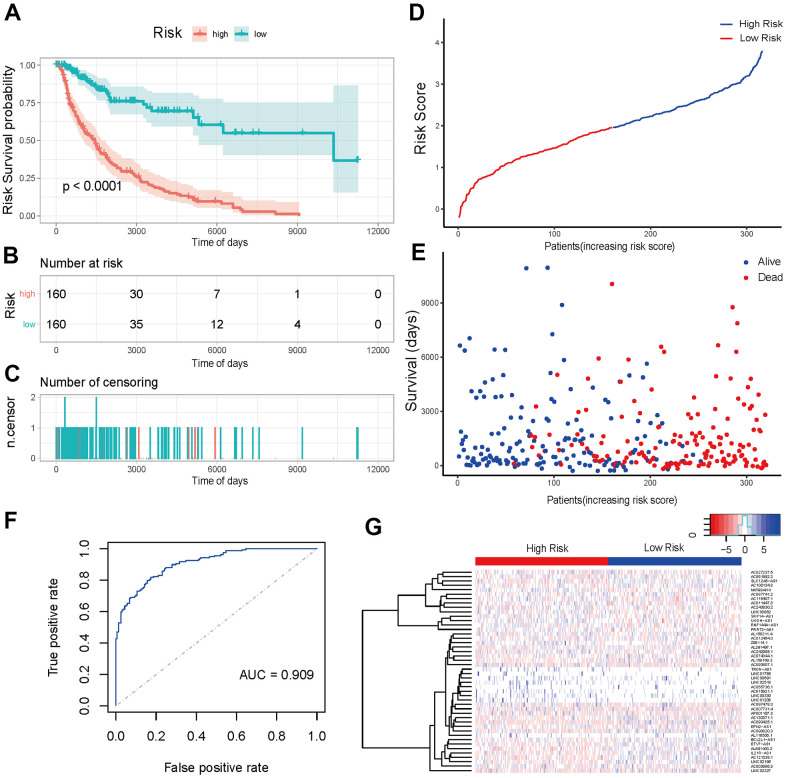
**Prediction effectiveness analysis and verification of prognosis risk scoring model.** (**A**) K-M curve of the high-risk group (red) versus low-risk group (green). (**B**) Sample risk table. (**C**) Sample censoring diagram. (**D**) The distribution of high-risk group (blue) and low-risk group (red) based on the risk score ranking. (**E**) Scatter plots of survival duration and status of high-risk group and low-risk group. The horizontal axis was the sample, and the vertical axis was the survival time. The blue dots represented survival and red represented death, respectively. (**F**) The ROC curve of the training sets. The grey dotted line was the random line, the blue curve was the AUC curve. (**G**) The heat map of lncRNAs expression in high- and low-risk groups. The red and blue bars represented the low-risk group and the high-risk group. And the evolution from red to blue represented the expression level of genes. The bluer zones indicated higher expression while the redder zones indicated lower expression. The horizontal coordinates of Figure 6D, 6E represented samples with increasing risk score. The 160 samples on the left were low-risk group, and the 160 samples on the right were high-risk group.

The K-M analysis was first performed for high- and low-risk groups in training sets using survival data to verify the prediction effectiveness of the model. The K-M curve showed that the low-risk group had a highly significant positive impact on survival (p-value < 0.001) (Figure 6A). Risk scores and survival duration and statue of each sample suggested that the prognosis of patients in the low-risk group was obviously better than that of patients in the high-risk group (Figure 6D, 6E). All the above results indicated that the model had a brilliant prediction effectiveness. In addition, the model was further used to predict the survival of the samples through the predict function, and the ROC curve was drawn. The ROC curve revealed that the AUC value was 0.909, which manifested that the model was highly precise (Figure 6F). The heat map of lncRNAs expression in high- and low-risk groups was shown in Figure 6G.

### External data set validation of prognosis model

**Figure 7:**
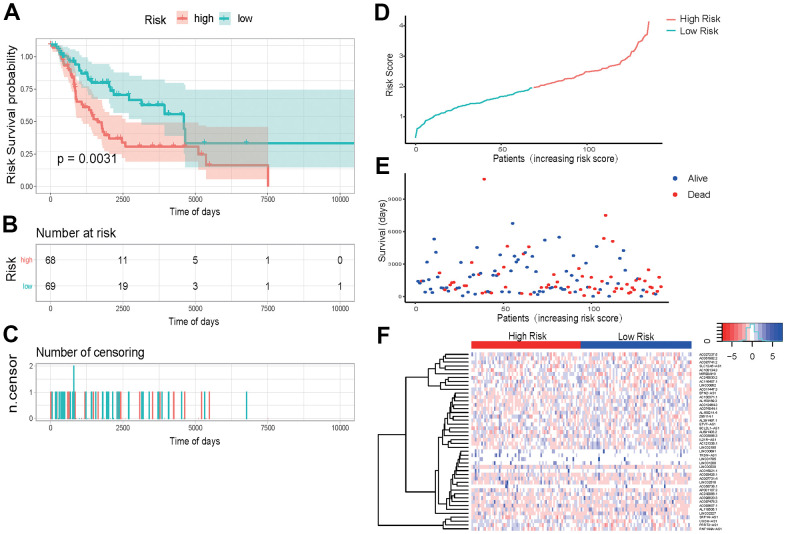
External data set validation of prognosis model (**A**) K-M curve of the high-risk (red) versus low-risk (green) group. (**B**) Sample risk table. (**C**) Sample censoring diagram. (**D**) The distribution of high-risk group (red) and low-risk group (green) based on the risk score ranking. (**E**) Scatter plots of survival duration and status of high-risk group and low-risk group. The horizontal axis was the sample, and the vertical axis was the survival time. The blue dots represented survival and red represented death, respectively. (**F**) The ROC curve of the testing sets. The grey dotted line was the random line, the blue curve was the AUC curve. (**F**) The heat map of lncRNAs expression in high- and low-risk groups. The red and blue bars represented the low-risk group and the high-risk group. And the evolution from red to blue represented the expression level of genes. The bluer zones indicated higher expression while the redder zones indicated lower expression. The horizontal coordinates of Figure 6D, 6E represented samples with increasing risk score. The 69 samples on the left were low-risk group, and the 68 samples on the right were high-risk group.

The prediction effectiveness of this model for prognosis of SKCM patients was further validated in the 137 testing sets. In the K-M curve, the influence of high- and low-risk group on survival reached an extremely significant level (P-value < 0.0031) (Figure 7A). Risk scores and survival duration and statue of each sample suggested that the prognosis of patients in the low-risk group was obviously better than that of patients in the high-risk group (Figure 7D, 7E). These results indicate that our model can effectively predict prognosis of SKCM patients.

### Immune cell infiltration analysis between high- and low-risk groups

**Figure 8:**
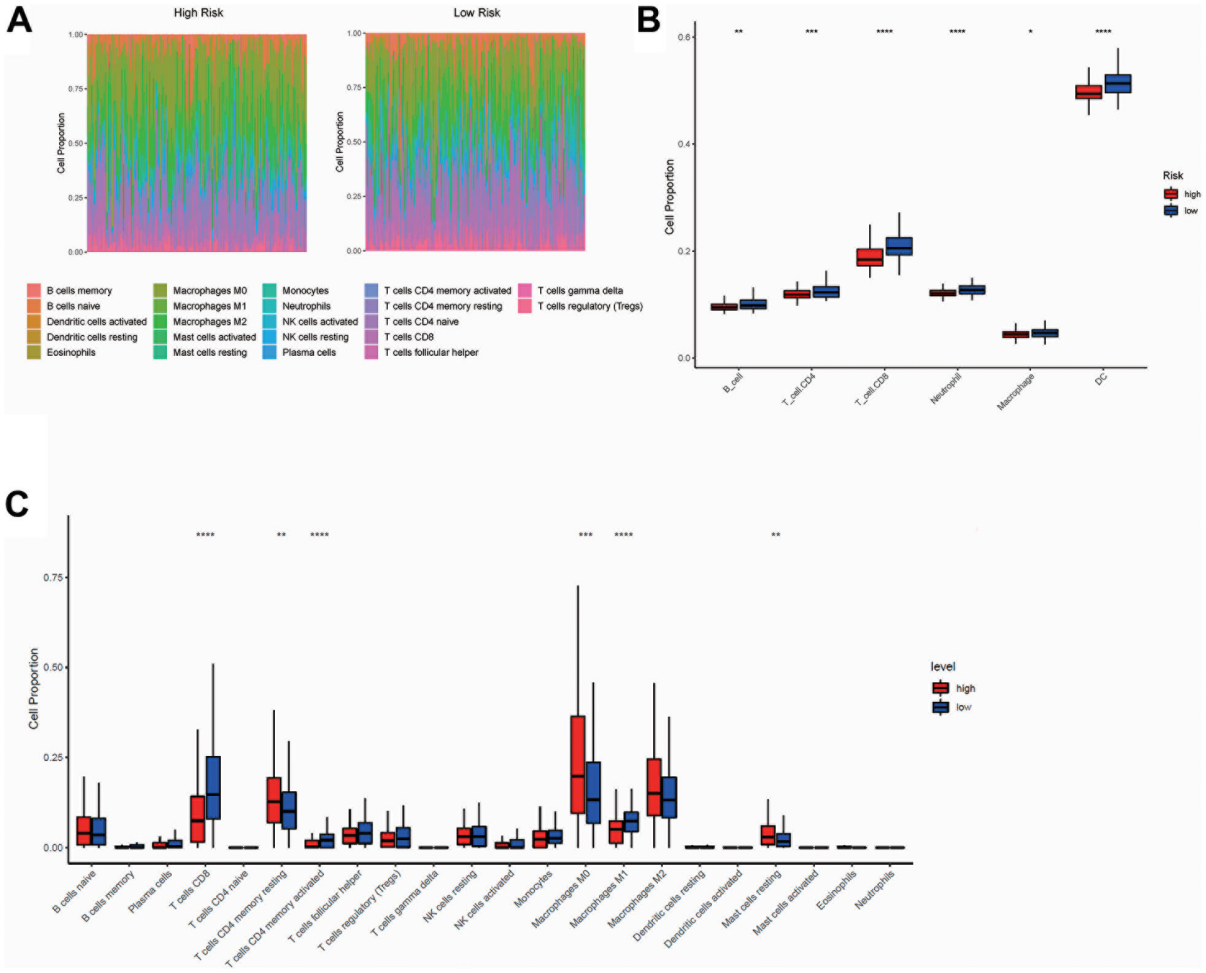
**Immune cell infiltration analysis between high- and low-risk group.** (**A**, **C**) The proportions of 22 immune cells infiltrated in the high- and low-risk groups from CIBERSORT analysis. (**B**) The proportions of B cells, CD8+ T cells, CD4+ T cells, neutrophils, macrophages, and dendritic cells infiltrated in the high- and low-risk groups from TIMER analysis. The horizontal axis of Figure B, C was the immune cells, and the vertical axis was the cell infiltration proportion. High-risk and low-risk groups were marked in red and blue, respectively.

Since the model was established based on immlncRNA, in order to further explore the relationship between risk score and tumor immune microenvironment, immune cell infiltration analysis was performed using TIMER and R packet CIBERSORT algorithms. CIBERSORT analysis showed the proportions of 22 immune cells infiltrated in the high- and low-risk groups (Figure 8A, 8B). The overall proportion of T-cell subtypes was higher in the low-risk group, while the overall proportion of macrophage subtypes was higher in the high-risk group. But the B cell subtypes did not differ significantly between the two subgroups. While the results of TIMER analysis showed that the proportions of B cells, CD8+T cells, CD4+ T cells, neutrophils, macrophages, and dendritic cells were all higher in the low-risk group (Figure 8C).

### Stability analysis of the model

**Figure 9:**
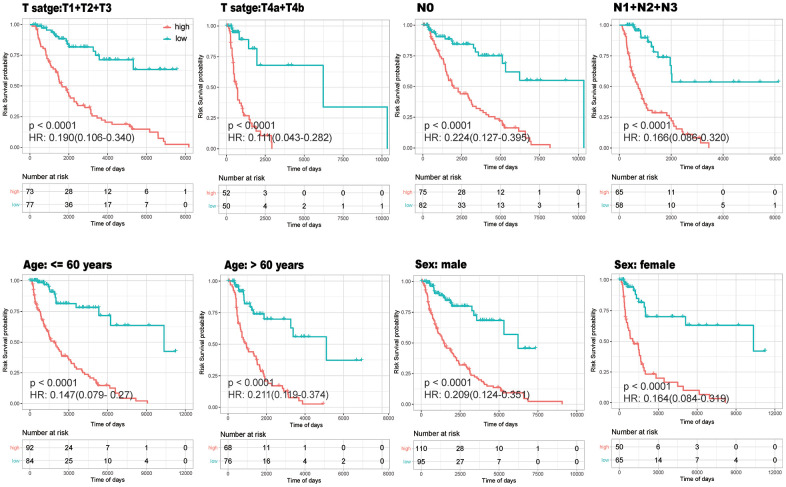
**Stability Analysis of the model.** K-M curves drawn within different subgroups according to high- (red) and low-(green)risk groups to verify whether the model was stable in different age, gender and tumor condition.

In order to verify if the model was stable in different age, gender and tumor condition, univariate Cox regression analysis of survival data and risk grouping was performed within the different subgroups. The K-M curves for the different subgroups showed that survival was consistently lower in the high-risk group than in the low-risk group (P<0.0001) (Figure 9). This further indicates that the established model is relatively stable and has a brilliant prediction effectiveness regardless of the distribution of T stage, N stage, age and gender in the sample.

### Univariate and multivariate Cox regression analysis verification

**Figure 10:**
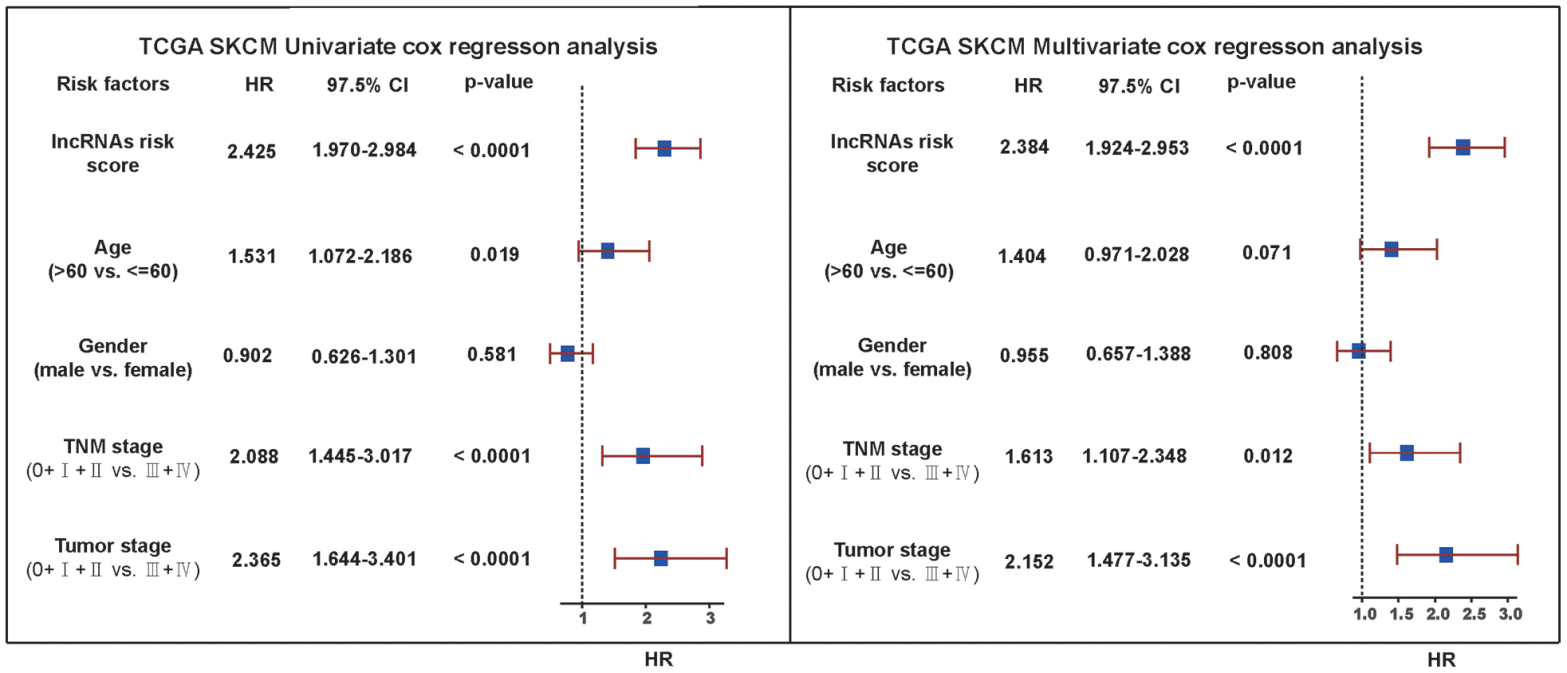
**Univariate and multivariate Cox regression analysis verification.** Univariate and multivariate Cox analysis results for other factors affecting SKCM including lncRNA risk score, age, gender, TNM stage and tumor stage. The vertical dotted line represented HR=1. We considered HR>1 as a survival disadvantage and HR<1 as a survival advantage.

To further explore if risk score could be used as an independent factor for prognostic prediction and to verify the stability of the model application, univariate and multivariate Cox regression analysis was performed for other factors affecting SKCM. Firstly, four possible SKCM influencing factors, including age, gender, TNM stage and tumor stage were selected from phenotypic data, and 5 factors were obtained by adding lncRNA risk score. Next, univariate Cox regression analysis was performed for the 5 factors with survival data alone (Figure 10). Results demonstrated that lncRNA risk score had the strongest correlation with SKCM, Hazard ratio was the highest, and the correlativity was significant (HR = 2.425, 95% CI = 1.970–2.984, P <0.0001). Moreover, TNM stage (HR = 2.088, 95%CI = 1.445–3.017, P <0.0001) and tumor stage (HR = 2.365, 95%CI = 1.644–3.401, P <0.0001) also visibly related with SKCM. However, the 97.5% CI (Confidence) interval of these two factors is relatively larger. It implies that the prediction results based on these two factors may be unstable. Additionally, the correlation between age (HR = 1.531, 95%CI = 1.072–2.186, P =0.019), gender (HR = 0.902, 95%CI = 0.626–1.301, P =0.581) and SKCM was weak, especially for gender factors, the correlativity was not significant. The above factors were further integrated to carry out multivariate Cox regression analysis. The results showed that lncRNA risk score remained the strongest correlation with SKCM, Hazard ratio was the highest, and the correlativity was significant (HR = 2.384, 95%CI = 1.924–2.953, P <0.0001). Hazard ratio was also high in TNM stage (HR = 1.613, 95%CI = 1.107–2.348, P <0.0001) and tumor stage (HR = 2.152, 95%CI = 1.477–3.315, P <0.0001). From multivariate Cox regression analysis, we also found that Hazard ratios of all factors were reduced compared with univariate Cox regression analysis. Therefore, a combination of some important factors may produce a better prognostic effect, while the prognostic effect of single factors is relatively unstable.

### Enrichment analysis via Co-lncRNA

**Figure 11:**
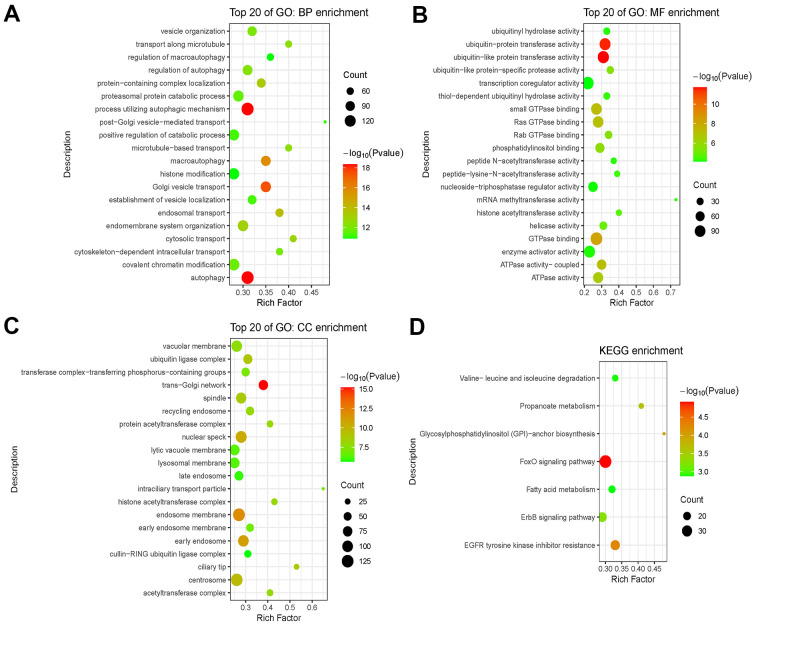
**Functional enrichment analysis of mRNAs co-expressed with lncRNAs.** (**A**–**C**) GO analysis including BP enrichment analysis (**A**), CC enrichment analysis (**B**), MF enrichment analysis (**C**). (**D**) KEGG pathway enrichment analysis. Abbreviations: GO: Gene Ontology; BP: Biological Process; CC: Cellular Component; MF: Molecular Function; KEGG: Kyoto Encyclopedia of Genes and Genomes.

Eventually, functional enrichment analysis was applied to explore the possible role of the 46 immlncRNA in the immune regulation of SKCM. A total of 2997 Co-expressed mRNAs of 46 lncRNAs in the model were extracted from the Skin (TCGA SKCM Pathologic Stage) database of Co-lnRNAs. Among them, only 4 lncRNAs had significantly correlated co-expressed mRNAs, including SRP14-AS1, RNF144A-AS1, MIR99AHG, UGDH-AS1. These mRNAs were further analyzed by GO enrichment analysis and KEGG enrichment analysis (Figure 11). BP enrichment mainly obtained intracellular transport and autophagy and other related pathways (Figure 11A). CC enrichment mainly obtained Golgi bodies, vacuoles and other organelles as well as acetyltransferase complex pathways (Figure 11B). MF enrichment mainly obtained ubiquitin protein transferase activity, GTPase activity, acetyltransferase activity and other pathways (Figure 11C). KEGG enrichment mainly obtained protein degradation related and fatty acid metabolism related pathways (Figure 11D). These results are consistent with the immune response process, suggesting that related lncRNAs may play an important role in the immune regulation of SKCM.

## DISCUSSION

SKCM is one of the most aggressive skin malignancies [1]. The prognosis is poor due to early metastasis, which is the main cause of death [2]. Therefore, early detection of SKCM and stratification of risk assessment are essential to treat SKCM and to improve survival rate. As one of the most immunogenic tumors, the role of immune regulation and immunotherapy of SKCM are always the central issues [16]. Meanwhile, in recent years, more and more studies have shown that lncRNA plays an important role in TNM staging, tumor invasion and metastasis, and prognosis of SKCM [12, 23]. Therefore, the exploration of immlncRNA in SKCM will further reveal the formation mechanism of SKCM and lay a solid foundation to improve the prognostic effect. In this study, based on 471 cancer samples extracted from the TCGA database and 555 normal samples extracted from the GTEx database, we found that 721 DE-immlncRNAs were significantly correlated with SKCM prognosis. We constructed an immlncRNA-related prognostic risk score model based on 46 immlncRNAs, which had significant correlations with prognosis, and effectively differentiated the SKCM into the high- and low-risk groups. Our analyses clearly indicate that this model has accurate and stable predictive efficiency. Furthermore, we also conducted immune cell infiltration analysis and immlncRNAs function enrichment analysis, and the results showed that there were differences in immune cell infiltration between the high- and low-risk groups, and immlncRNAs were related to a variety of immune response process signaling pathways, suggesting that relevant immlncRNAs may play an important role in the immune regulation of SKCM.

A few of the 46 selected immlncRNAs have been reported to be associated with the progression and prognosis of other tumors. MIR99AHG (also known as MONK), for example, highly expresses in acute megakaryocytic leukemia (AMKL) cell lines, and MONK knockout impacts the proliferation of leukemia cell lines and inhibits the growth of AMKL cancer samples [24]. Moreover, it is reported that MIR99AHG expression is up-regulated in gastric cancer, which is associated with clinical progression and poor prognosis of gastric cancer [25]. MIR99AHG is also used to build prognostic risk score models in many tumors, including head and neck squamous cell carcinoma, lung squamous cell carcinoma, breast cancer, and carcinogens [26–28]. All these suggest that MIR99AHG is a potential tumor biomarker that can be used for prognostic assessment and to guide targeted therapy. However, there have been no reports on its role and mechanism in SKCM, so further research is needed. LINC00691 has also been confirmed to be highly expressed in gastric cancer patients, which simultaneously promotes the expression of epithelial growth factor to accelerate the proliferation and invasion of gastric cancer cells [29]. RT-qPCR detection and survival analysis from more than 100 clinical samples reveal that the expression of LINC00691 is also up-regulated in non-small cell lung cancer, which is associated with poor prognosis [30]. Upregulation of LINC00691 is also found in renal cell carcinoma [31]. PRRT3-AS1 is a novel lncRNA that has been shown to be associated with the proliferation of prostate cancer cells. PRRT3-AS1 silencing will inhibit the proliferation of prostate cancer cells and promote apoptosis and autophagy [32]. In addition, PRRT3-AS1 regulates glioblastoma cells proliferation and metastasis through MAPK signaling pathways [33]. A recent study uncovers that PRRT3-AS1 is associated with poor prognosis of hepatocellular carcinoma [34]. This also suggests that PRRT3-AS1 may play an important role in the proliferation of cancer cells and is associated with poor prognosis of cancer. The specific roles of these immlncRNAs and the others of 46 immlncRNAs in the SKCM are worthy of further experimental verification and study, which will greatly contribute to understanding formation and development mechanism of SKCM and target immunotherapy. It also lies a more solid foundation for the prognosis assessment and improvement of the prognosis of patients.

To further explore the specific relationship between immlncRNA and immune regulation and the mechanisms of immlncRNA in SKCM biological processes, we performed immune cell infiltration analysis using TIMER and R packet CIBERSORT algorithms. Both algorithms indicated that there were significant differences in immune cell infiltration in high- and low-risk groups. Considering the different calculation methods of the two algorithms and the difference in the prediction sensitivity of different cells, the results of the two algorithms are inevitably different. But for key cells, such as CD8^+^T cells, the results obtained by the two algorithms were consistent, that is, in the low-risk group, the proportion of CD8^+^ T cells infiltrated was significantly increased. This is consistent with previous findings that T cell infiltration increases survival in SKCM patients and is associated with better prognosis [35]. Moreover, studies have confirmed that CD8^+^T cells can infiltrate into tumor tissue sites and kill tumor cells [36]. Tumor-associated macrophages (TAM), however, can promote the metastasis and invasion of tumor cells to other sites through the contact with tumor cells and molecular transfer [37]. Therefore, our results clearly reveal that the imbalance of T cell and macrophage ratio will significantly affect the prognosis and the response to immunotherapy. The functional enrichment analysis of the selected immlncRNA also reveals that the signal pathway is involved and is consistent with the immune response, which confirms that immlncRNA may play an important role in the immune regulation of SKCM.

The 46 immlncRNAs screened can be used as brilliant tumor markers, which can be used to create a prognostic model, develop immunotherapy, provide more research directions and possibilities for the immune regulation mechanism of SKCM. Herein, all our conclusions are based on bioinformatics analysis. In our next step, we will select several important immlncRNA to further verify and understand true mechanisms by *in vitro* and *in vivo* experiments. In addition, in order to correlate the obtained data with clinical practice, we retrieved the top 150 differentially expressed genes according to high- and low-risk groups and put them into the Connectivity Map (CMAP) database in an attempt to screen potential small molecule drugs related to SKCM. Taking q-value < 0.05 as the threshold, we finally obtained 3 small molecule drugs with certain therapeutic potential. They are PCO-400, AM-251 and talipexole (Supplementary Table 1). These drugs may provide therapeutic targets for future research.

In conclusion, we successfully constructed a 46 immlncRNA-related prognostic risk score model with excellent predictive efficacy. Different prognoses of high- and low-risk groups were associated with certain immlncRNA expression imbalance, different immune cell infiltration, and multiple immune reactive-related signaling pathways. Our study provides a good way to evaluate the clinical prognosis of SKCM, and also provides more possibilities for the research on the immune regulation mechanism and immunotherapy of SKCM.

## CONCLUSIONS

In summary, we successfully constructed a 46 immlncRNA-related prognostic risk score model with excellent predictive efficacy. Different prognoses of high- and low-risk groups were associated with certain immlncRNA expression imbalance, different immune cell infiltration, and multiple immune reactive-related signaling pathways. Our study provides a good method for the clinical early diagnosis and prognostic judgment of SKCM, and also provides more possibilities to investigate the immune regulation mechanisms and to develop immunotherapy of SKCM.

## MATERIALS AND METHODS

### Flow chart of analysis plans

**Figure:**
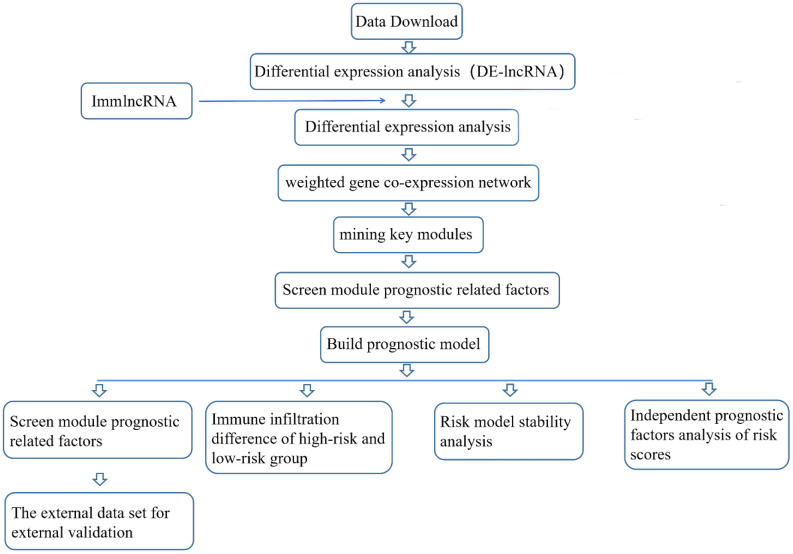


### Data download

1. Download SKCM expression data from UCSC TCGA database:
  https://gdc.xenahubs.net/download/TCGA-SKCM.htseq_counts.tsv.gz; Full metadata simultaneous acquisition of Phenotypic data and Survival data for each sample
2. Download normal human expression data from GTEx database and extract expression matrix:
  https://toil.xenahubs.net/download/gtex_gene_expected_count.gz; Full metadata
3. Access immlncRNA related information from immLnc database:
http://bio-bigdata.hrbmu.edu.cn/ImmLnc/
4. Download human gtf file (Homo_sapiens.GRCh38.99.gtf.gz) from Ensembl database and access information of lncRNA and symbol:
http://www.ensembl.org/info/data/ftp/index.html

### Skin cutaneous melanoma (SKCM) differential expression analysis

Differential expression analysis LncRNAs were obtained by R package edgeR. The forms of expression matrix in two databases were both log_2_^(x+1)^. Then the counts were drawn by round(2^a-1). Due to batch effects between TCGA cancer samples and GTEx samples, the standardized expression profile was obtained by TMM normalization method built in edgeR. Furthermore, the differential expression genes were obtained by threshold value of abs (log_2_FC) > 1 and FDR < 0.05. The expression matrix used in subsequent analysis was standardized expression profile.

### Integration of DE-lncRNAs

Venn diagrams of DE-lncRNAs and immlncRNA were plotted using R package VennDiagram. Shared DE-immlncRNAs were integrated by merge formula for subsequent WGCNA.

### Weighted gene co-expression network analysis (WGCNA)

Weighted gene co-expression network analysis was performed on DE-immlncRNAs via R package WGCNA, which obtained the strongest correlativity module with tumor for subsequent analysis.

### Univariate Cox regression analysis of lncRNAs in key modules

To deeply mine lncRNAs related to tumor in modules, we further extracted lncRNAs from cancer samples. As shown in Table 1, combined with survival data (samples without existent survival data were excluded), we used R-package survival and survminer to carry out batch Cox single-factor regression analysis and K-M curve drawing, respectively. After regression analysis, significant lncRNAs were screened with P < 0.05 as the threshold for subsequent lasso regression analysis.

**Table 1:** Survival data statistics.

	**Event=0**	**Event=1**
Samples	235	222

### Establishment of immlncRNAs prognosis risk scoring model via lasso regression analysis

Lasso regression was used to reduce the dimension of the significantly correlated lncRNAs obtained in the previous step, and construct risk scoring model, which mainly depended on the R-package glmnet. To build more accurate regression models, lambda screening was carried out by cross validation. Then we selected models corresponding to lambda.min. The expression matrix of related genes in the model was further extracted, and the risk score of each sample was calculated by the following formula. The samples were divided into high-risk group (High Risk) and low-risk group (Low Risk) by using median as a cutoff for later model validation.


$${\text{RScore}}_{\text{i}}=\sum_{\text{j}=1}^{\text{n}}{\text{exp}}_{\text{ji}}\times {\text{β}}_{\text{j}}$$

### Prediction effectiveness analysis and verification of prognosis risk scoring model

According to the above analysis, the high- and low-risk groups were obtained. Combined with the survival data, the K-M curve was drawn, and p-value < 0.01 was judged as significant model construction, then the predict function in R was further used to predict the results, and the AUC value of the model was calculated, and then the ROC curve was drawn with AUC > 0.8 to judge the accuracy of the model construction.

### External data set validation of prognosis model

Because there were lack of lncRNAs-related data sets, and no suitable external data sets were found, we cut data via R package caret and obtained training sets possessing about two-thirds of the total data and testing sets possessing rest of the total data. We calculated the risk scores of the testing set. Similarly, the data were divided into high-risk group and low-risk group. Then according to survival data, we plotted K-M curves and got significant P-value (p-value<0.01), which identified this model with better predictive effects than others.

### Immune cell infiltration level analysis of high-risk group and low-risk group was calculated through TIMER and CIBERSORT algorithm

CIBERSORT algorithm can infer the proportion of 22 immune cells in the sample according to the expression of some genes. Firstly, the expression data of characteristic genes were extracted from the complete expression data to obtain expression matrix of characteristic genes. Then, combined with the existing immune cell signature file, the proportion of immune cells in different groups was calculated by using R-package CIBERSORT. At the same time, box plots were formed by Wilcoxon rank sum test to facilitate the comparison of 22 immune cell infiltration levels between the low-risk group and the high-risk group. TIMER can also analyze immune cell infiltration. TIMER 2.0 (https://cistrome.shinyapps.io/timer/) is a platform for analyzing the infiltration of immune cells in tumor tissues based on RNA-Seq expression profiling data. It mainly provides the infiltration of six types of immune cells including B cells, CD4^+^ T cells, CD8^+^ T cells, neutrophils, macrophages and dendritic cells. We uploaded the expression information and analyzed the immune cell infiltration in high- and low-risk groups on this platform.

### Stability analysis of the model

In order to verify whether the model was stable in different age, gender and tumor condition, we divided training set samples into different groups according to the method of Table 2 (samples without relevance were excluded), used univariate Cox regression analyses in groups and plotted K-M curves of high-risk group and low-risk group. By calculating whether p-value was significant, the model could be proved to be stable.

**Table 2:** Sample classification method of model validation verification.

**T stage**	**N stage**	**Age**	**Sex**
T1+T2+T3 (150)	N0 (157)	>60 years (176)	Male (205)
T4a+T4b (102)	N1+N2+N3 (123)	<=60 years (144)	Female (115)

### Univariate and multivariate Cox regression analysis verification

To verify whether risk scores were independent prognostic factors, we performed univariate regression analyses on other factors in the training set. Other factors included age, gender, TNM (tumor node metastasis classification) staging and tumor stage. To ensure consistency with follow-up analysis, all samples were required to contain the information mentioned above. Then we had multivariate Cox regression analyses on overall prognosis of above 5 factors to prove that the prognostic effect of risk score was more significant than other factors.

### Enrichment analysis via Co-lncRNA

Since there are only protein-coding gene functions in GO (Gene Ontology) and KEGG (Kyoto Encyclopedia of Genes and Genomes) functional database, we needed networking with lncRNA and mRNA. By analyzing and searching for mRNA co-expression with lncRNA, Co-lncRNA database built co-expression networks between mRNA and lncRNA. In addition, Co-lncRNA database studied the function via GO and KEGG of co-expressed mRNA. The web of the database: http://bio-bigdata.hrbmu.edu.cn/Co-LncRNA/.

Co-expressed mRNA from lncRNAs in the model was used from Skin (TCGA SKCM pathologic stage). Expression identification chose linear regression calculation, coef>2 and p-value<0.001. Then, functional annotations were performed via R package clusterProfiler.

### Availability of data and materials

The data used to support the findings of this study are available.

## Supplementary Material

Supplementary Table 1
