# Supplementary material for: Skin cutaneous melanoma properties of immune-related lncRNAs identifying potential prognostic biomarkers
Source: Aging (Albany NY). 2022 Mar 31;14(7):3030–48. doi: 10.18632/aging.203982 (PMC9037265; doi:10.18632/aging.203982)
Supplement: Supplementary Table 1 [file aging-14-203982-s001.pdf]

SUPPLEMENTARY TABLE

Supplementary Table 1. List of small molecule drugs with some therapeutic potential for SKCM.

pert_iname	cell_iname	pert_type	pert_idose	pert_itime	moa	target_name	raw_cs	fdr_q_nlog10	norm_cs
PCO-400	VCAP	trt_cp	10 uM	6 h	Potassium channel activator	KCNJ8|ABCC9	-0.58	15.65	-1.78
AM-251	A549	trt_cp	10 uM	6 h	Cannabinoid receptor antagonist	CNR1|GPR18|GPR55	-0.58	15.65	-1.8
talipexole	VCAP	trt_cp	10 uM	6 h	Adrenergic receptor agonist|Dopamine receptor agonist	DRD2|HTR3A|ADRA2A	-0.56	1.45	-1.72
